# Active Transport of the Ubiquitin Ligase MID1 along the Microtubules Is Regulated by Protein Phosphatase 2A

**DOI:** 10.1371/journal.pone.0003507

**Published:** 2008-10-24

**Authors:** Beatriz Aranda-Orgillés, Johanna Aigner, Melanie Kunath, Rudi Lurz, Rainer Schneider, Susann Schweiger

**Affiliations:** 1 Max-Planck Institute for Molecular Genetics, Berlin, Germany; 2 Institute of Biochemistry, University Innsbruck, Innsbruck, Austria; 3 Division of Pathology and Neuroscience, Ninewells Hospital, University of Dundee, Dundee, United Kingdom; Temasek Life Sciences Laboratory, Singapore

## Abstract

Mutations in the MID1 protein have been found in patients with Opitz BBB/G syndrome (OS), which is characterised by multiple malformations of the ventral midline. MID1 is a microtubule-associated protein that stabilizes microtubules and, in association with the regulatory subunit of protein phosphatase 2A (PP2A), α4, provides ubiquitin ligase activity for the ubiquitin-specific modification of PP2A. Using Fluorescence Recovery After Photobleaching (FRAP) technology, we show here that MID1 is actively and bi-directionally transported along the microtubules, and that this movement is directly linked to its MAP kinase and PP2A-mediated phosphorylation status. Intact transport depends on both kinesins and dyneins and is inhibited upon colcemide treatments. MID1 proteins carrying missense mutations in the α4 binding domain still bind the microtubules but cannot be actively transported. Likewise, knock-down of the α4 protein, inhibition of PP2A activity by okadaic acid and fostriecin or the simulation of permanent phosphorylation at Ser96 in MID1 stop the migration of MID1-GFP, while preserving its microtubule-association. In summary, our data uncover an unexpected and novel function for PP2A, its regulatory subunit α4 and PP2A/α4/mTOR signaling in the active transport of the MID1 ubiquitin ligase complex along the cytoskeleton. Furthermore, a failure in the microtubule directed transport of this protein complex would be an attractive mechanism underlying the pathogenesis of OS in patients with B-box1 mutations.

## Introduction

Patients with Opitz BBB/G syndrome (OS) are characterised by a diverse spectrum of ventral midline malformations. The most characteristic symptoms are hypertelorism, dysphagia and hypospadias. Cleft lip and palate, agenesis of the corpus callosum, tracheo-esophageal fistulas, congenital heart defects and anal defects are found additionally with variable penetrance [1]. Migration and ventral invasion of neural crest cells and epithelial-mesenchymal transition are the two leading mechanisms during the development of the affected ventral midline structures [reviewed in [2]].

The X-linked form of the syndrome is caused by mutations in the *MID1* gene, which codes for the MID1 protein, a member of the RBCC protein family [3]. The protein is characterised by an N-terminal tripartite motif consisting of a RING finger, two B-boxes and a coiled-coil domain and a C-terminus containing a fibronectin Type III [4] domain, a COS domain [5] and a B30.2 domain [reviewed in [6]]. Mediated by the B-box1 domain, MID1 interacts with the regulatory subunit of protein phosphatase 2A (PP2A), the α4 protein [7], which is the mammalian homologue of the yeast protein TAP42 [reviewed in [8]]. In this protein complex, MID1 has ubiquitin ligase activity and thereby regulates the ubiquitin-specific modification and proteasomal degradation of the microtubule-associated catalytic subunit of PP2A (PP2Ac). Additionally, recent results indicate a novel function of the MID1 complex in RNA-binding and localized translation [9].

MID1 is a microtubule-associated phospho-protein with microtubule-stabilizing properties [10]. Its dephosphorylation is mediated by PP2A, which is targeted towards MID1 by α4. It has been suggested, that microtubule-association of MID1 is regulated by its mitogen activated protein kinase (MAPK) and PP2A dependent phosphorylation at position serine 96 (S96) [11], which is located in the linker region between RING finger domain and B-Box1 domain.

Most mutations found in OS patients cluster in the C-terminus of the protein [1], [3], [12]–[17] and lead to the loss of microtubule-association of these proteins [10]. Accordingly, connection between the ubiquitin ligase MID1/α4 and microtubule-associated PP2Ac is disrupted, the enzyme accumulates and microtubule-associated proteins are hypophosphorylated [7]. Mutations in the B-box1 domain disturb the interaction with α4 and accordingly with microtubule-associated PP2Ac (BA et al., unpublished data).

In this report, we show that GFP-tagged MID1 protein is bi-directionally transported along the microtubules, and that this movement depends on microtubule-integrity and on kinesin and dynein motor proteins. It is abolished when the MID1 protein carries mutations in the B-box1 domain, in cells with α4 loss-of-function, after inhibition of PP2A activity and after exchanging serine 96 into either glutamic (E) or aspartic acid (D), both simulating permanent protein phosphorylation. Interestingly, microtubule-association of MID1 is not influenced by any of these mutations or treatments. In conclusion, our data present a novel function of PP2A and its regulatory subunit α4 in the microtubule mediated transport of the MID1 protein complex. Furthermore, by showing interference of mutations in the B-Box1 domain of MID1 with its microtubule-associated transport, they suggest an attractive mechanism underlying the pathogenesis of OS in patients with such mutations.

## Results

### Bi-directional transport of MID1-GFP along the microtubules

**Figure 1:**
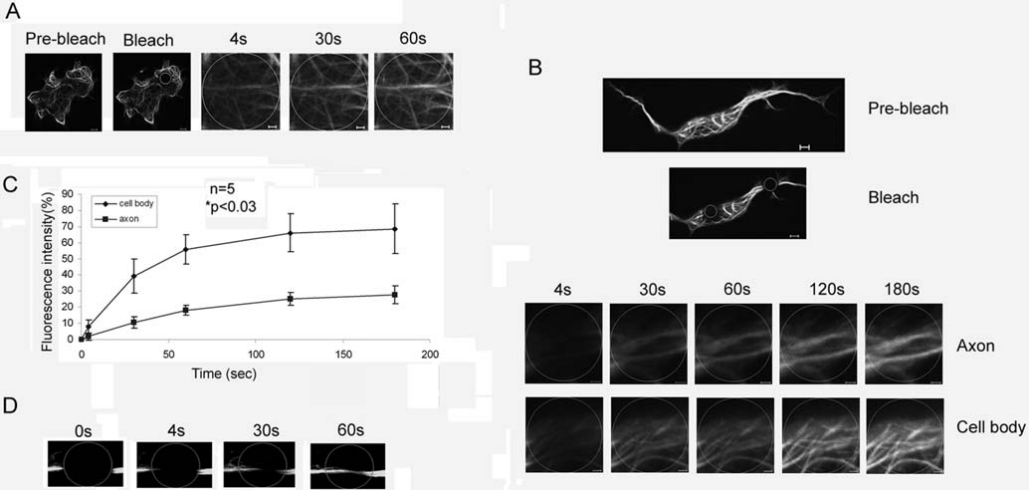
Recovery of MID1-GFP induced green fluorescence after photobleaching (FRAP). Cells before bleaching (pre-bleach) and extensions of selected areas at different time-points after bleaching (1 sec, 4 sec, 30 sec and 60 sec in A; 4 sec, 30 sec, 60 sec, 120 sec and 180 sec in B; 0 sec, 4 sec, 30 sec and 60 sec in D) are shown. Scale bars in overview pictures represent 5 µm, scale bares in scale up pictures are 1 µm. A) Recovery of fluorescence in a cell body of HeLa cells. B) Fluorescence recovery in in the cell body and the axon of an F11 cell. C) Statistical evaluation of recovery rates seen in B relative to time after bleaching. D) Fluorescence recovery from both directions (periphery and cell body) in the axon of an F-11 cell.

Many microtubule-associated proteins have been shown to move along the microtubules. In order to study migration of the ubiquitin ligase MID1 along the microtubules, we transfected HeLa cells with GFP-tagged MID1 (MID1-GFP) and analysed them in a laser-scan microscope for FRAP. As described previously [10], [18], wild-type MID1-GFP showed a defined microtubule-associated pattern. Full recovery of the GFP signal after bleaching was obtained within a few seconds (30–60 sec, Fig. 1a). Similarly fast recovery was observed in cell-bodies of F11 cells, a hybrid cell line of mouse neuroblastoma cell line N18TG-2 and embryonic rat dorsal-root ganglion (DRG) neurons (Fig. 1b), while fluorescence returned significantly more slowly in axons (60–120 sec, Fig. 1b, c). Detailed analysis of the recovery of the green signal in axons demonstrated recovery from the centrosome and from the periphery suggesting a bi-directional mechanism (Fig. 1d).

### Transport of MID1-GFP depends on intact microtubule-dynamics

**Figure 2:**
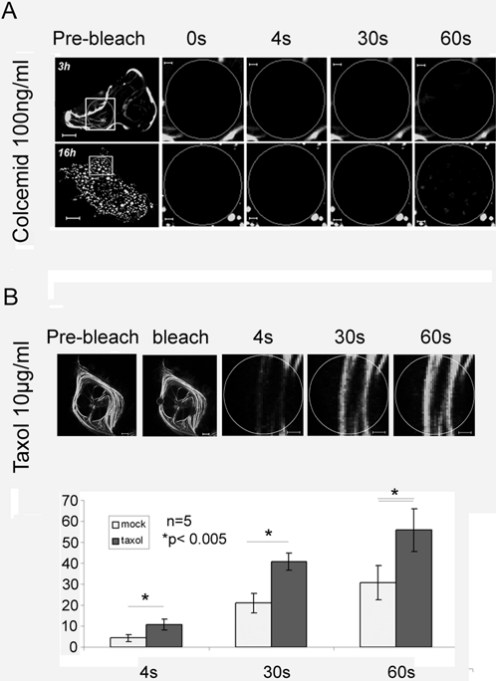
Recovery of MID1-GFP induced green fluorescence after photobleaching depends on microtubule integrity. Whole cells before bleaching (pre-bleach) and extensions of selected areas at different time-points after bleaching are shown. Scale bars in overview pictures represent 5 µm, scale bares in scale up pictures are 1 µm. A) FRAP in cells treated with 100 ng/ml colcemid over 3 or 16 hours. B) FRAP in cells treated with 5 µg/ml taxol over 5 hours.

Microtubule-dependency of the transport of MID1-GFP was further analysed in HeLa cells treated with drugs that interfere with microtubule dynamics. While a few bundles were still left after 3 hours, all microtubules were destroyed after treatment with 100 ng/ml colcemide over a period of 16 hours. As shown in figure 2A, after 16 h, apart from few green dots resulting from protein diffusion in the cell, no fluorescence recovery of the MID1-GFP signal was observed even after 180 seconds. By contrast, treatment of the cells for 5 hours with 5 µg/ml taxol, a drug that stabilizes microtubules (Fig. 2b), significantly accelerated fluorescense recovery indicating that the transport of MID1-GFP relies on intact microtubule-dynamics.

### Dyneins and kinesins are involved in the transport of MID1-GFP

**Figure 3:**
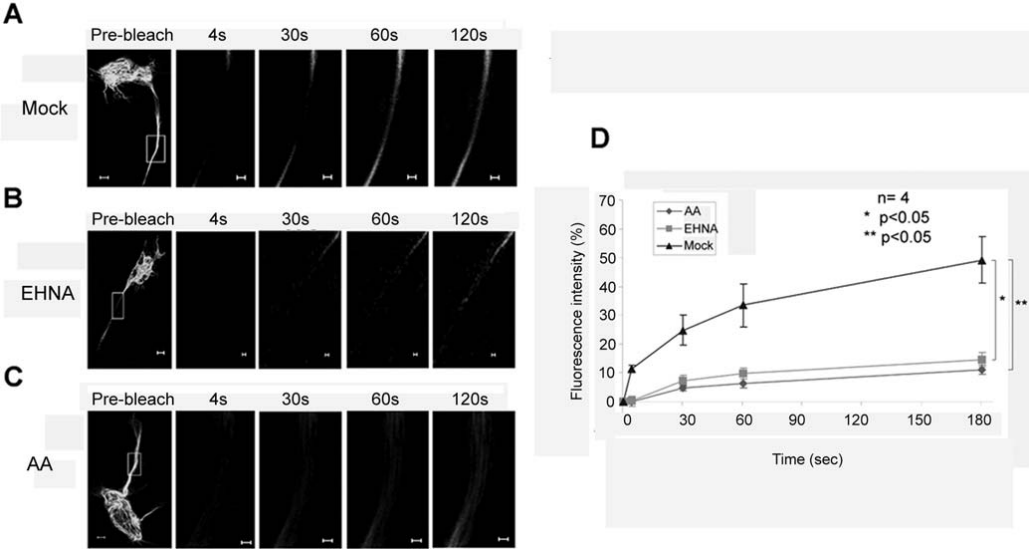
Recovery of MID1-GFP induced green fluorescence after photobleaching depends on dynein and kinesin motor proteins. Whole cells before bleaching (pre-bleach) and extensions of selected areas at different time-points after bleaching are shown. Scale bars in overview pictures represent 5 µm, scale bares in scale up pictures are 1 µm. A) FRAP in a mock treated F-11 cell. B) Retrograde FRAP is inhibited in an F-11 cell that was treated with 1 mM of the dynein inhibitor Erythro-9-(2-Hydroxy-3Nanyl) Adenine (EHNA) for 30 min. C) Significant slow-down of FRAP in an F-11 cell after treatment with 10 µM of the unspecific kinesin inhibitor aurintricarboxylic acid (AA) for 30 min. D) Statistical evaluation of recovery rates seen in A-C relative to time after bleaching. Four cells of each experiment have been analysed (n = 4). P values are given.

Two different classes of molecules are known to actively transport proteins along the microtubules. While kinesins transport towards the plus ends of microtubules and therefore the cell periphery, dyneins are adjusted to the minus ends, which locates at the organizing centre of microtubules. As suggested from the bi-directional transport seen in axons of F11 cells (see above), inhibition of both molecule classes significantly influences the recovery rate of the MID1-GFP signal. Treatment of cells with 10 mM of Erythro-9-(2-Hydroxy-3-Nonyl)Adenine (EHNA), an inhibitor of dynein activity [19], led to inhibition of retrograde transport and fluorescence recovery only took place from the centrosome (Fig. 3b and d). On the other hand, treatment of cells with aurintricarboxylic acid, an inhibitor of kinesin activity [20], resulted in a slow-down of recovery from both directions as compared to the mock-treated control (Fig. 3a, c and d). Both analyses were performed in axons.

### Mutations in the B-box1 impede transport of MID1-GFP along the microtubules

**Figure 4:**
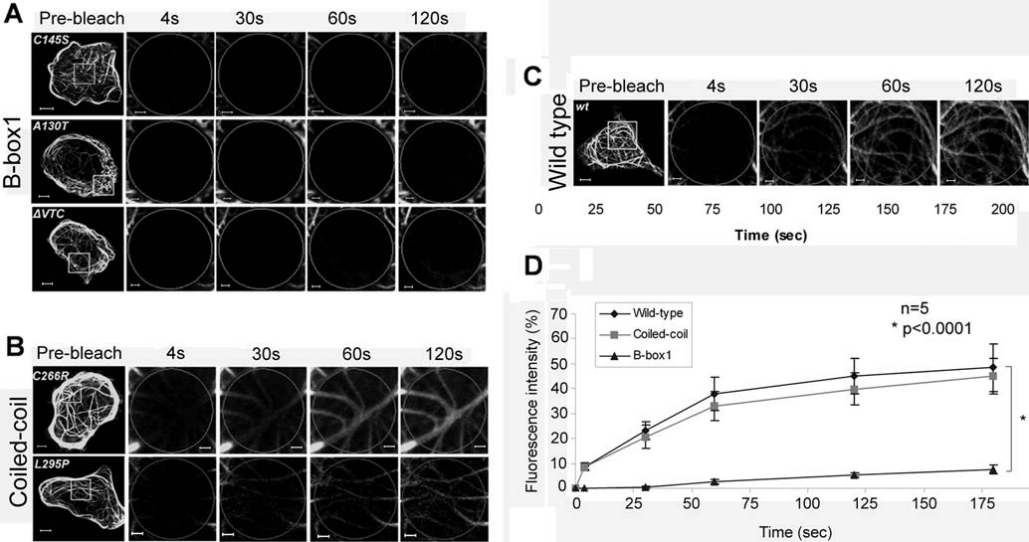
Recovery of green fluorescence induced by MID1-GFP mutants after photobleaching. Whole cells before bleaching (pre-bleach) and extensions of selected areas at different time-points after bleaching are shown. Scale bars in overview pictures represent 5 µm, scale bares in scale up pictures are 1 µm. A) FRAP of three different MID1-GFP proteins carrying mutations in the B-Box1 domain of MID1 (C145S, A130T and ΔVTC in HeLa cells. B) FRAP of two different MID1-GFP proteins carrying mutations in the coiled-coil domain of the MID1 protein (C266R and L295P) in HeLa cells. C) FRAP of wild-type MID1 in HeLa cells as control. D) Statistical evaluation of recovery rates seen in A-C relative to time after bleaching. Five cells of each experiment have been analysed (n = 5). P-value is given.

Various mutations in the MID1 proteins have been identified in OS patients [1], [3], [10], [12]–[17]. Most of those are located in the C-terminus of the protein and interfere with its binding to microtubules [10]. Apart from these, missense mutations in the B-box1 domain of the MID1 protein have been identified [1], [14]. Proteins carrying these mutations still bind to microtubules (BA et al., manuscript in preparation), but fail to bind the α4 protein [7] (BA et al., manuscript in preparation). We have used MID1-GFP proteins carrying three different mutations in this domain, C145S , A130T (both patients identified in this study, see Methods section) and ΔVTC [13] for FRAP analysis (Fig. 4a). For comparison, we analysed two mutations in the coiled-coil domain, C266R [13] and L295P [1], which also do not affect microtubule binding of the protein (Fig. 4b). Interestingly, while the last two showed fluorescence recovery comparable to the wild-type (Fig. 4b, c and d), no recovery was seen with either of the B-Box1 mutations (Fig. 4a and d), suggesting that functional B-box1 domain is required for the correct transport of MID1 at the microtubules.

### α4 and PP2A activity are necessary for the transport of MID1-GFP

**Figure 5:**
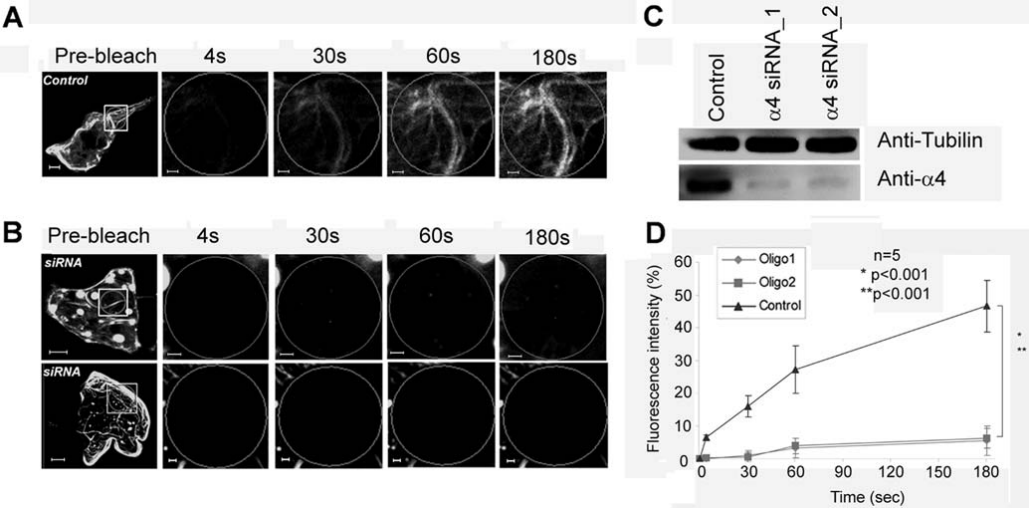
Recovery of MID1-GFP induced green fluorescence after photobleaching depends on the α4 protein. Whole cells before bleaching (pre-bleach) and extensions of selected areas at different time-points after bleaching are shown. Scale bars in overview pictures represent 5 µm, scale bares in scale up pictures are 1 µm. A) FRAP of MID1-GFP in a HeLa cell transfected with non-silencing siRNA oligonucleotides. B) FRAP of MID1-GFP in a HeLa cell transfected with two different (upper and lower panel) siRNA oligonucleotides for α4 specific knock-down. C) Western blot with lysates of HeLa cells transfected with non-silencing siRNAs control or with one (α4 siRNA oligo 1) or the other (α4 siRNA oligo 2)of the a4 specific siRNAs. Blot was incubated with a specific anti-α4 antibody. Equal loading is demonstrated with an anti-tubulin antibody. D) Statistical evaluation of recovery rates seen in A and B relative to time after bleaching. Five cells of each experiment have been analysed (n = 5). P-values are given.

Since MID1 interacts with α4, and thereby with PP2A, through its B-box1 domain, the previous FRAP data pointed towards a functional role for α4 and PP2A in the microtubule-associated transport of the MID1 protein. In order to confirm this, we knocked down α4 with specific RNAi oligonucleotides and analysed these HeLa cells for fluorescence recovery of MID1-GFP. Confirming our hypothesis, no recovery could be observed in cells with α4 knock-down (Fig. 5b and d) even after 180 seconds , while the recovery rate in cells treated with non-silencing siRNAs (control, Fig. 5a and d) was comparable to the wild-type. Efficiency of the knock-down is shown on a Western-blot using an anti-α4 antibody (Fig. 5c). Interestingly, while tubulin staining showed no changes in microtubules organization in cells with α4 knock-down compared to cells treated with nonsilencing siRNAs (data not shown), microtubule association of MID1-GFP seemed to be slightly disturbed after α4 knock-down (Fig. 5b).

**Figure 6:**
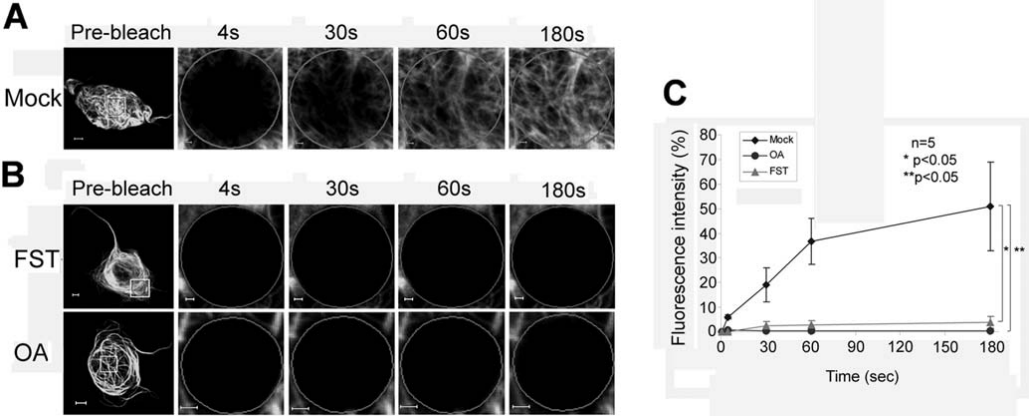
Recovery of MID1-GFP induced green fluorescence after photobleaching depends on PP2A activity. Whole cells before bleaching (pre-bleach) and extensions of selected areas at different time-points after bleaching are shown. Scale bars in overview pictures represent 5 µm, scale bares in scale up pictures are 1 µm. A) FRAP of MID1-GFP in a mock-treated HeLa cell. B) FRAP of MID1-GFP in HeLa cells pre-treated with either 50 nM of the unspecific PP2A inhibitor okadaic acid (OA) for 30 min (upper panel) or with 200 nM of the specific PP2A inhibitor fostriecin for 30 min (lower panel). C) Statistical evaluation of recovery rates seen in A and B relative to time after bleaching. Five cells of each experiment have been analysed. P-values are given.

Similarly, treatment of cells with the PP2A inhibitors okadaic acid (OA) and fostriecin (FST) led to a complete inhibition of fluorescent recovery (Fig. 6b and c). Full recovery after 30–60 seconds was seen in the mock-treated control (Fig. 6a and c), while no recovery was seen after OA and FST treatment after 180 seconds, once again indicating a central involvement of PP2A activity in the microtubule-associated transport of MID1-GFP.

**Figure 7:**
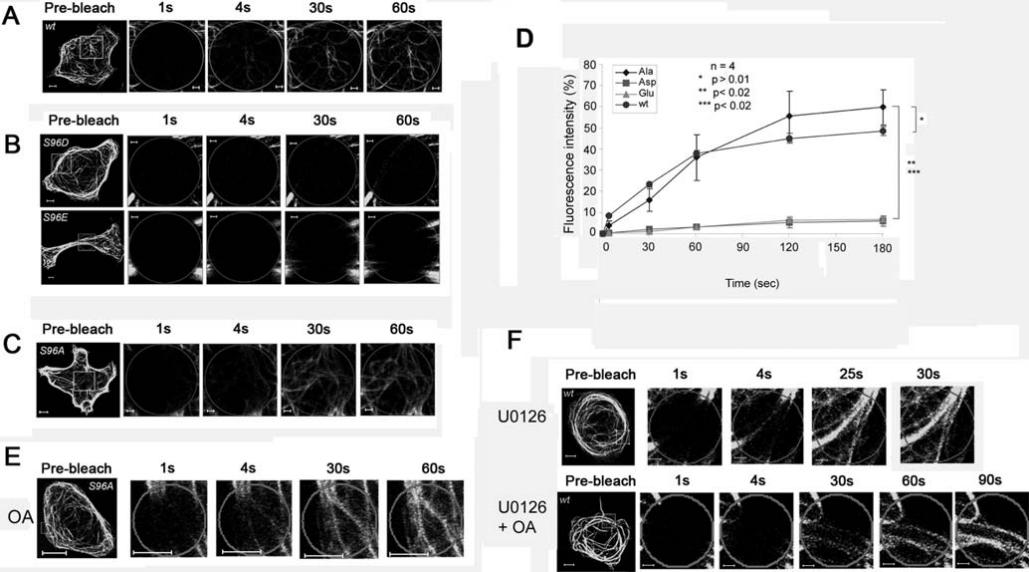
Recovery of MID1-GFP induced green fluorescence after photobleaching depends on serine 96. Whole cells before bleaching (pre-bleach) and extensions of selected areas at different time-points after bleaching are shown. Scale bars in overview pictures represent 5 µm, scale bares in scale up pictures are 1 µm. A) FRAP of wild-type MID1-GFP in a HeLa cell. B) FRAP of MID1-GFP proteins carrying a mutated S96, S96D (upper panel) and S96E (lower panel), in HeLa cells. C) FRAP of MID1-GFP carrying a mutated S96, S96A, in a HeLa cell. D) Statistical evaluation of recovery rates seen in A-C relative to time after bleaching. Five cells of each experiment have been analysed. P-values are given. E) The S96A mutant does not react on OA treatment; fluorescence recovers similar to the untreated wild-type MID1-GFP when treating cells that express the S96A mutant with OA. F) Cells expressing wild-type MID1-GFP show recovery of the fluorescence comparable to the wild-type when treated with the MAPK inhibitor U0126 (upper panel) or pretreated with UO126 and subsequently treated with OA (lower panel).

It has been suggested previously that MAP kinase and PP2A regulate the phosphorylation status of MID1 on serine 96. Therefore, its PP2A dependent dephosphorylation could be necessary for proper microtubule-associated transport along the microtubules. To test this hypothesis, we produced three different point mutations on serine 96. Two of them, S96D and S96E, brought negative charges and thereby simulated continuous phosphorylation (Fig. 7b), whereas S96A completely inhibited phosphorylation (Fig. 7c). As expected, while S96A showed recovery rates comparable to the wild-type (Fig. 7a, c and d), neither S96D nor S96E were transported or showed fluorescent recovery even after 180 seconds (Fig. 7b and d). In contrast to the wild-type, transport of the phosphorylation free S96A mutant along the microtubules was not inhibited by okadaic acid treatment prior to analysis (Fig. 7e). Also, cells overexpressing wild-type MID1-GFP that were pretreated with the MAPK inhibitor UO126 did not react on okadaic acid treatment, confirming a functional role of MAPK dependent phosphorylation in the regulation of the transport of the MID1-GFP protein along the microtubules (Fig. 7f). Surprisingly, contrary to previously suggested data [11], all S96 mutant proteins showed undisturbed microtubule-associated pattern. Also, UO126 treatment did not interfere with microtubule-association of the wild-type protein (Fig. 7f).

## Discussion

Active transport of molecules from the cell centre towards the cell periphery and back, as shown for the ubiquitin ligase MID1 in this study, is very important for cell function and cell survival in the embryo. For example, establishment of asymmetry in the Drosophila oocyte and the early embryo depends on the transport of proteins and RNA along the microtubules [reviewed in [21]]. Furthermore, microtubules act as tracks to deliver microtubule plus end-binding proteins to the leading edge of a polarizing cell in a kinesin-mediated manner [reviewed in [22], [23]]. Following protein recruitment, Rac1 is being activated, lamellipodia are formed and polarised cell-migration is possible [reviewed in [23], [24]]. Migration of neural crest cells into ventral structures is essential during the development of the ventral midline [reviewed in [2]]. Mutations in the microtubule-associated MID1 protein lead to defective establishment of these structures and the development of the ventral midline disorder OS [3]. We show here that, while wild-type MID1 is being transported bi-directionally along the microtubules, proteins carrying mutations in the B-box1, that have been found in OS patients still bind to the microtubules, but do not move anymore. Together with the OS phenotype and the specific structures involved, these data point at a MID1 function that is closely related with microtubule dependent polarisation and migration of neural crest cells.

Interestingly, we have seen a significantly slower transport of MID1 along axonal microtubules than in the cell body. This could reflect energy and motor protein supply gradients in the cell with high concentration in the cell body close to the mitochondria and at the microtubule-organizing centre and lower concentrations in the cell peripherie. However, we find an increase in transport speed after taxol treatment which suggests that MID1 transport speed is in direct proportion with the stability of the microtubules. Higher transport speeds close to the microtubule-assembly centre where most stable microtubules are found would be a logical consequence.

Continuous outward polymerisation of microtubules from the microtubule organizing center (MTOC) towards the cell periphery has been demonstrated in the polarised, migrating cell [reviewed in [25]]. This makes the transport of proteins supporting microtubule polymerisation to the tip of an outgrowing microtubule an important factor. MID1 has been shown to stabilize microtubules [10] and, as shown here, its transport along the microtubules depends on kinesin motor proteins. These observations would suggest functional similarities to Par-1 which, by also having microtubule stabilizing properties [26], is a key organizer of microtubule arrays important for the polarised transport of cell polarity [reviewed in [27]]. Accordingly, dysfunctional transport of MID1 in migrating neural crest cells in patients with OS would lead to a disequilibrium of stabilizing and destabilizing microtubule-associated proteins (MAPs) at the tip of microtubules growing out towards the leading edge of the cell and could result in either a slow down of migration or in the misdirection of the cell.

Interestingly, we have observed not only kinesin but also dynein-dependent transport of MID1. Similarly, both kinesin and dynein dependent transport in neurons has been shown for Par-3, another member of the Par-protein family that are widely conserved regulators of cell polarity and asymmetry [[28], [29], reviewed in [27]]. Reasons for the retrograde, dynein mediated transport of MID1 could either be a feedback regulation at the outgrowing tip in order to avoid an excess of microtubule-stabilizing factors which would perturb the delicate equilibrium of microtubule dynamics. Additionally it could allow rapid recycling of the MID1 protein for functions at the centrosome. However, it is striking that, while the dynein inhibitor EHNA completely stops retrograde MID1 transport, the kinesin inhibitor seems to decelerate both, kinesin-driven antegrade and dynein-related retrograde transport. This could reflect a lack of supply at the cell peripherie. When antegrade transport is blocked and only very limited possibilities are available to synthesize fresh protein in the cell peripherie, the density of molecules awaiting retrograde transport shrink, which would result in a slow down of retrograde transport.

Our data clearly show that by dephosphorylating S96, PP2A stimulates the transport of the MID1 ubiquitin ligase complex along the microtubules. For MID1, it has been demonstrated previously that it interacts with microtubules in a phosphorylation dependent manner [11]. Treatment of cells with mitogene activated protein kinase (MAPK) inhibitors led to a partial redistribution of MID1-GFP from the microtubules and co-expression of MID1-GFP with α4 resulted in a decrease of MID1 phosphorylation. It was further suggested that dephosphorylation of MID1 at S96 by α4-associated PP2A might reduce the association of MID1 with the microtubule network and result in cytoplasmic localisation. In this study, we have used three different S96 mutants, two of which simulate continuous phosphorylation at that position (S96D and S96E), while the third completely inhibits MID1 phosphorylation at S96 (S96A). Interestingly, all three MID1-GFP mutants showed similar microtubule-associated distribution as the wild-type (Fig. 7). Also, the MAPK inhibitor UO126 did not interfere with microtubule-association of wild-type MID1-GFP in our hands. Instead of phosphorylation dependency of microtubule-association, we found that the microtubule-associated transport of MID1 heavily relies on the PP2A-mediated dephosphorylation of its N-terminus. We further show that this dephosphorylation is closely linked to α4, the mammalian homologue of the yeast protein TAP42. TAP42, α4 and their association to PP2A is regulated by the target-of-rapamycin (TOR) pathway and by rapamycin [30]–[33], which has been related to microtubule-associated protein transport [34] and cell migration [35] before. Our data therefore not only show a novel role of PP2A in microtubule-associated protein transport, but also bring up additional evidence that the TOR/mTOR pathways is involved in the regulation of intracellular protein transport, localized stabilisation of microtubules and cell migration.

In summary, our data provide evidence that dysfunctional microtubule directed and PP2A dependent transport of the microtubule stabilizer MID1 is an important pathomechanism underlying the ventral midline disorder Opitz BBB/G syndrome. They further show that association to the regulatory PP2A subunit α4 is essential for this transport and that point mutations in the α4 binding domain completely destroy the protein's cellular mobility.

## Materials and Methods

### Constructs

Two novel mutations found in OS patients have been used: One patient presented with hypertelorism, broad nasal bridge, strabismus, cleft lip and palate, hypospadias and small ears with a right pre-auricular pit. He was found to have a de novo 388G>A mutation in MID1, predicting an A130T change in the B-box1 domain. The second patient had hypertelorism, down-slanting palpebral fissures, broad nasal bridge, posteriorly rotated ears, cleft lip and palate, and hypospadias. He was found to harbour a de novo 433T>A MID1 mutation, predicting a C145S change in the B-box1 domain.

In-vitro mutagenesis experiments were performed on MID1 in the pEGFP-C1 vector (MID1-GFP) [10] and according to the instruction manual provided with the QuickChange® Site Directed Mutagenesis Kit (Stratagene).

### MID1-GFP Transfection and Live Cell Imaging

1×10^5^ HeLa per well of a 6-well plate were grown for 24 hours on glass cover slips and transfected with 1 µg plasmid DNA using 5 µl of lipofectamine (Invitrogen) according to the manufacturer's instructions.

2×10^5^ F11 cells per well of a 6-well plate were grown for 24 hours in Ham's F-12 medium supplemented with 10% FCS on glass coverslips and were transfected using 5 µl of lipofectamine and 8 µl of Plus Reagent (Invitrogen) in 2 ml OptiMEM. After 2 h medium was changed to Ham's F-12 containing 18% FBS. Medium was changed again after 3 h to Ham's F-12 supplemented with 10% FBS.

Fluorescence recovery after photobleaching (FRAP) analysis of MID1-GFP was carried out on cultured F11 neurons and HeLa cells. Transfected cells were randomly selected and bleached for between 0.5 and 1.1 s using 100% power from a 25 mW argon ion laser. Recovery was imaged at low laser power, and cells were examined for ∼4 min, with imaging approximately every 2 sec. Percent recovery was determined by subtracting arbitrary average background values outside the cell from average values from the whole cell or selected subregions, then dividing this by the difference in fluorescence intensity of the selected region before bleaching and the average background values (I_spot_−I_bkgd_ (post-bleach)/I_spot_−I_bkgd_ (pre-bleach). Each of the traces was calculated from an average percent recovery of n = 5 cells at each time point, starting at 4 s post bleach.

Series of 50–100 single section images were collected with the help of a time series programme. For imaging, the laser power was attenuated to 2% of the bleach intensity. Images were acquired on an LSM 510 (Carl Zeiss, Jena, Germany) with the Planapochromat 63-/1.4 objective. EGFP fluorescence was detected using the 488 laser line of an argon laser (25% of 25-mW nominal output) in conjunction with a LP 505 filter. All live cell imaging was done at room temperature. Images were analysed with the Zeiss LSM image examiner software and Image J.

### Cell Treatment

20–24 h after transfection, cells were treated for 30 min with 50 nM okadaic acid (Sigma), 10 µM U0126 (Sigma), 200 nM fostricien (Sigma), 10 µM aurintricarboxylic acid (Calbiochem) or 1 mM Erythro-9-(2-Hydroxy-3-Nonyl) Adenine (Calbiochem). In addition, cells were treated with 100 ng/ml colcemid (Biochrom) for 3 and 16 h or 5 µg/ml taxol (Sigma) for 5 h. Cells were washed with PBS and analysed under a LSM 510 microscope as described above.

### siRNA Transfection

1×10^5^ HeLa cells were seeded in a 6-well plate 24 hours before transfection. Cell were incubated with a solution containing 1.5 µg DNA, 3 µl of siRNA oligo (α4 oligonucleotide 1: sense CGUUGCUAUGGCAUCUCAAdTdT/antisense UUGAGAUGCCAUAGCAACGdTdT, α4 oligonucleotide 2: sense GUACCUUUUGGUGCCAGCGdTdT/antisense CGUUGGCACCAAAAGGUACdTdT, non-silencing oligonucleotide: sense UUCUCCGAACGUGUCACGUdTdT/antisense ACGUGACACGUUCGGAGAAdTdT, Qiagen), 2 µl dreamfect (Biosiences), and 100 µl of OptiMEM (Sigma) in 2 ml of medium without antibiotics. Cells were incubated at 37°C in a humidified incubator with 5% CO2 v/v for 72 h.

### Western Blot

200 µg of proteins were loaded on a 12% SDS-page, blotted on a PVDF membrane (Roche) and incubated with a specific anti-α4 antibody (Trockenbacher et al., 2001) at 4°C overnight. For loading control, the same blot was incubated with an anti-tubulin antibody.
